# Editorial: Seed Microbiome Research

**DOI:** 10.3389/fmicb.2022.943329

**Published:** 2022-07-08

**Authors:** Wilfried Jonkers, Pedro E. Gundel, Satish Kumar Verma, James Francis White

**Affiliations:** ^1^Bejo Zaden B.V, Warmenhuizen, Netherlands; ^2^Centro de Ecología Integrativa, Institute of Biological Sciences, University of Talca, Talca, Chile; ^3^Instituto de Investigaciones Fisiológicas y Ecológicas Vinculadas a la Agricultura (IFEVA), National Scientific and Technical Research Council (CONICET), Facultad de Agronomía, Universidad de Buenos Aires, Buenos Aires, Argentina; ^4^Department of Botany, Institute of Science, Banaras Hindu University, Varanasi, India; ^5^Department of Plant Biology, Rutgers, The State University of New Jersey, New Brunswick, NJ, United States

**Keywords:** seed microbiome, seed endophytes, germination, vertical transmission, beneficial microorganisms

With sequencing technologies, we are able to detect viruses, bacteria, and fungi that are outside (ectosphere) or inside (endosphere) of plants. Accordingly, there is a boom in articles exploring factors that control the plant associated microbiome by specifically looking at different plant organs/tissues across species, and different biotic and abiotic environmental conditions. Viewing the plant together with the associated microorganisms as a supra-organism (i.e., holobiont) presumes that symbionts play functional roles in determining host phenotype (Zilber-Rosenberg and Rosenberg, 2008; Vandenkoornhuyse et al., 2015). However, while there are plant-microorganism systems in which the functional roles of symbionts are well-established (Rodriguez et al., 2009; White et al., 2019), the fact is that most of the studies today are descriptive, predominantly determining which microbes are present.

Members of the plant microbiome can be taken from the soil (Walsh et al., 2021), or can come within—or attached to—the seed (Truyens et al., 2015; Shade et al., 2017; Berg and Raaijmakers, 2018). Plant-microorganism symbiosis can present different degrees of coevolution and so, stability, and degree of integration (Ellers et al., 2012). Some symbiotic systems have evolved to a point that the microorganism/s is/are conserved across maternal linages by means of vertical transmission (i.e., microorganisms pass from mother plant to seeds) (Gundel et al., 2011; Abdelfattah et al., 2021). There are conditions that lead symbiosis with free-living microorganisms to evolve into stable mutualistic interactions (see Thompson, 2005; Ellers et al., 2012). Vertical transmission of microorganisms may result in evolution of mutualisms because fitness of both partners is tightly aligned (Ewald, 1987; Herre et al., 1999; Gundel et al., 2008). For instance, the association of certain grasses (Family: Poaceae) with vertically transmitted *Epichloë* fungal endophytes is labeled as a defensive mutualism since fungal alkaloids confer resistance against herbivory (Rodriguez et al., 2009; White and Torres, 2009; Panaccione et al., 2014). Therefore, besides identifying the variables controlling the plant microbiome, we want to understand the functions these microorganisms play in the evolution of plant phenotype (Gundel et al., 2017; White et al., 2019).

Manipulation of the symbiotic states of plants is a breeding strategy in agriculture (Gundel et al., 2013; Wei and Jousset, 2017). In some programs, different fungal endophyte strains are inoculated to improve forage cultivars and turf grasses, a strategy that relies on the persistence in—and transmission through, the seed (Johnson et al., 2013). Spraying plants with beneficial microorganisms at flowering has been proposed as a technical strategy for manipulating the seed microbiome and add agricultural desirable traits in crops (Mitter et al., 2017). However, the flowering stage is also critical for pathogen infection, which once in the seed, will affect the next generation of plants. It is interesting then, to understand the effect of the seed microbiome on the infection rates and transmission of phytopathogenic microorganisms (Barret et al., 2016).

In this special topic, we focused on seed microbiomes because: (i) they may be critical for seed germination and seedling establishment, (ii) they may affect the ecology and evolutionary dynamics of plant symbiosis by connecting the maternal environment with that of offspring, (iii) they may be critical pathways for pathogenic diseases to spread, and (iv) seed microbiomes represent a way to manipulate beneficial symbionts in agriculture. One paper (Chandel et al.) highlights the storage strategy implications on seed microbiomes in seed vaults and another two (Thomas and Saha; Thomas et al.) reveal the presence of “cytobacts” (bacteria that enter into plant cells) in multiple plant species. Three papers (von Cräutlein et al.; Bastías et al.; Laihonen et al.) deal with fungal endophytes in the genus *Epichloë*, which associate with grasses forming a systemic and asymptomatic symbiosis. Three other articles (Kim and Lee; Johnston-Monje et al.; Kumar et al.) involve the seed microbiome and microbial endophytes for seedling growth, development, and nutrient acquisition. Two other articles (Parmar et al.; Redman et al.) focus on the association of plant health regarding abiotic and biotic stress and associated microbes.

Fungal endophytes in the genus *Epichloë*, associate with grasses forming a systemic and asymptomatic symbiosis. These endophytes grow in the apoplast of host aerial tissues producing alkaloids that protect plants against herbivores. While most of these endophytes are interspecific hybrids that reproduce asexually by growing hyphae in developing seeds (vertical transmission), some haploid species conserve the capacity of reproducing sexually and transmit both vertically and horizontally. An important volume of research shows that these endophytes alter the ecology and evolution of grass populations. In this special issue, von Cräutlein et al. were able to infer underlying processes to the population structure of *Epichloë festucae* Leuchtm., Schardl & M.R. Siegel (a haploid endophyte) in the host species *Festuca rubra* L. (Red Fescue) combining different molecular approaches. Their results suggest that sexual reproduction is important in southern European populations while asexual reproduction and vertical transmission seem to prevail in northern populations (von Cräutlein et al.). There was also a great variation in alkaloid genes but, interestingly, the variation within populations in this variable was lower in northern populations (von Cräutlein et al.). In common garden experiments, Laihonen et al. showed that the seed transmitted *E. festucae* also affected the ecological interaction of *F. rubra* plants with pathogens and herbivores. Interestingly, they found that plants associated with *E. festucae* were more susceptible to be infected by the biotropic pathogen *Claviceps purpurea* (Fr.) Tul.; but the presence of the pathogen was associated with a lower incidence of aphids (*Sitobion* sp.) due to a higher content of ergot alkaloids (Laihonen et al.). Bastías et al. tested the hypothesis that as a persistent and vertically transmitted endophyte, *E. occultans* (C.D.Moon, B.Scott & M.J.Chr.) Schardl controls the seed microbiome of *Lolium multiflorum* plants. In their experiment, *L. multiflorum* plants with and without endophyte were challenged by the aphid *Rhopalosiphum padi* and the microbiomes of the produced seeds were characterized by sequencing the bacterial 16S ribosomal RNA (rRNA) gene. *Epichloë* endophytes increased the bacterial diversity and affected the bacterial communities which were more equitable (all bacterial groups were equally abundant) than that of endophyte-free seeds (Bastías et al.). These three works illustrate how a vertically transmitted microorganism affects the ecology of host grasses by modulating the interaction with other microorganisms.

Several of the articles in this collection deal with seed-vectored bacteria or fungi. In a genomic study, Johnston-Monje et al. show that bacteria present on seeds colonize seedlings to dominate the seedling microbiome. This article and other studies underline the importance of the seed microbiome and microbial endophytes for seedling growth, development and nutrient acquisition (White et al., 2019). Using microscopy and metagenome profiling in a diverse set of plants, different cultivation-recalcitrant endophytic bacteria living intracellularly were found in many plant parts and deep in the seeds and embryos of watermelon [*Citrullus lanatus* (Thunb.) Matsum & Nakai] and grapevine [*Vitis vinifera* L.] (Thomas et al.). Many of these bacteria belong to the phylum Proteobacteria. This paper sheds a new light on intracellular endophytic bacteria as ubiquitous entities in vascular plants that can be transmitted vertically to the next generation. These authors refer to such intracellular bacteria as “cytobacs,” in reference to their presence in plant cell cytoplasm (see also White et al., 2019 for another discussion of these intracellular microbes). Thomas and Sahu examined bacteria transmitted within watermelon seeds and showed that bacteria transmitted in seeds could be seen within plant cells after seedlings form. An article by Kim and Lee examines diversity and distribution of both bacteria and fungi in seedlings and mature plants of rice. Redman et al. takes an ecological approach and shows that plant microbes in symbiosis with plants may drive expansion of coastal plants in the San Juan Archipelago of Washington State. Collectively, these papers suggest the importance of seed-vectored symbiotic microbes in facilitating plant growth, development, and ecological success.

Plants recruit diverse communities of microorganism from their surroundings and absorb them into their tissues as endophytes. These plant-associated microbes, or plant microbiota, play important roles in modulation of plant development (Berg and Raaijmakers, 2018; White et al., 2019). Although microbes have been isolated and reported from all parts of plants, occurrence of microbes in seeds is advantageous to plants because seed associated microbes may colonize the emerging seedlings to promote plant health (Verma et al., 2019; White et al., 2019). Composition and diversity of seed microbiota may be influenced by genotype, storage, and environmental conditions. Studies on influence of storage conditions on seed microbiota conservation have not been explored adequately. Chandel et al. showed that in soybean (*Glycine max* (L.) Merr.) initial seed drying before storage reduced the microbial composition. Further study suggested that storage of seeds at −20°C is standard for conservations of seed microbiota. Being an early colonizer of seedlings, seed endophytic microbes may provide stress tolerance against salt, drought, and heavy metals, and also may protect seedlings from soil pathogens during early stages of seedling development (Verma and White, 2018). Parmar et al. reported that a seed endophytic fungus *Epicoccum nigrum* improved Cd tolerance in *Dysphania ambrosioides* (L.) at all stages of its development. In another study of seed endophytic bacteria of pearl millet (*Pennisetum glaucum* L.), Kumar et al. found that seed endophytic bacteria are important during seedling development. They reported that removal of seed endophytic bacteria compromised the seedlings growth and increased susceptibility against *Fusarium* sp., however, when the same bacteria were re-inoculated, the seedlings restored their growth with reduced susceptibility against pathogens (Kumar et al.).

We hope this special issue sparks interest in seed microbiome research. We are in an unprecedented time in which we may study microbiomes and their effects on plant development and how interactions between microbes and plants are regulated. Seeds of plants are genetic resources that ensure plant propagation but also contain a plethora of microbes that may be used to secure healthy plants and agricultural soils.

## Author Contributions

All authors listed have made a substantial, direct, and intellectual contribution to the work and approved it for publication.

## Funding

PEG research was supported by the Agencia Nacional de Investigaciones Argentina (ANPCyT) PICT-2018-01593 and by the Fondo Nacional de Desarrollo Científico y Tecnológico (FONDECYT-2021-1210908). SKV obtained financial support from IoE BHU (incentive grant) and DBT project P-07/1265.

## Conflict of Interest

WJ is employed by company Bejo Zaden B.V. The remaining authors declare that the research was conducted in the absence of any commercial or financial relationships that could be construed as a potential conflict of interest.

## Publisher's Note

All claims expressed in this article are solely those of the authors and do not necessarily represent those of their affiliated organizations, or those of the publisher, the editors and the reviewers. Any product that may be evaluated in this article, or claim that may be made by its manufacturer, is not guaranteed or endorsed by the publisher.
